# Combined Effects of Compound Low-Sodium Alternative Salts and Vacuum Tumbling on the Quality, Water Distribution, and Microstructure of Marinated Beef

**DOI:** 10.3390/foods14040605

**Published:** 2025-02-12

**Authors:** Yanfeng Huang, Shujie Yang, Longtao Zhang, Song Miao, Zhiyong Xu, Baodong Zheng, Kaibo Deng

**Affiliations:** 1 Engineering Research Centre of Fujian-Taiwan Special Marine Food Processing and Nutrition, Ministry of Education, Fuzhou 350002, China; hyfeng0606@163.com (Y.H.); zlongtao@fafu.edu.cn (L.Z.); zbdfst@163.com (B.Z.); 2 College of Food Science, Fujian Agriculture and Forestry University, Fuzhou 350002, China; starysjj@163.com; 3 China-Ireland International Cooperation Centre for Food Material Science and Structure Design, Fuzhou 350002, China; 4 Teagasc Food Research Centre, Food Chemistry and Technology Department, Moorepark, Fermoy, Co., P61 C996 Cork, Ireland; 5 Fujian Yitai Food Development Co., Ltd., Putian 351100, China; cyy850222@163.com

**Keywords:** marinated beef, vacuum tumbling, low sodium, quality, water-holding capacity

## Abstract

This study proposes a compound low-sodium alternative salt (CLSAS) formulation (2.4% sodium chloride, 0.8% K lactate, 0.4% magnesium chloride, 0.4% Ca ascorbate, 0.2% L lysine, and 4% sorbitol) combined with vacuum tumbling for beef marination. The effects of 4% NaCl static marination (F), CLSAS static marination (L), and CLSAS vacuum tumbling (VT-L) on the physicochemical properties, water distribution, and microstructure of marinated beef were evaluated. Compared with F, L maintained similar yield and color, reduced cooking loss, and improved texture while lowering sodium content. VT-L further enhanced product yield, water content, color, texture, and tenderness. Both CLSAS and vacuum tumbling reduced the relaxation time of immobilized water, promoted orderly formation of protein structure, and altered the microstructure of myogenic fibers. VT-L additionally improved the water-holding capacity of myofibrils and further reduced the relaxation times of immobilized and free water. Overall, VT-L could be an effective approach for enhancing the quality of low-sodium meat products, providing a feasible basis for the industrial application of CLSAS for low-sodium marinated meat products.

## 1. Introduction

Marinated beef refers to a type of processed prepared dish that involves cutting, marinating, and packaging. Due to its convenience in cooking, prepared dishes align well with the demands of modern fast-paced lifestyles and hold significant market potential [1]. Marination is a crucial step in the processing of marinated beef [2]. This process utilizes osmotic pressure differences (immersion) or other mechanical forces (e.g., injection, tumbling, and ultrasound) to facilitate the penetration of the marinade into the muscle tissue [3,4,5,6]. The penetration of the marinade promotes the hydrolysis of muscle proteins, thereby improving the texture of meat products [7]. Additionally, it increases pH and reduces the isoelectric point of protein to enhance the water-holding capacity of the meat [8]. Sodium chloride (NaCl) is one of the most commonly used marinades due to its wide range effects on meat quality [9,10]. NaCl imparts saltiness to meat [11], enhances water-holding capacity [12], and improves the texture [13]. However, as the primary source of sodium in meat products, excessive NaCl intake (>5 g/day) has been associated with an increased risk of hypertension, cardiovascular disease, and coronary heart disease [14,15]. While NaCl plays a vital role in improving meat quality, its health implications have driven food scientists to focus on the development of low-sodium and healthy meat products [16,17].

Sodium salt substitutes, including potassium, calcium, and magnesium salts [18], are used in processed meat products to reduce sodium intake and lower the consequent health risks. Zhang et al. [19] demonstrated that magnesium chloride (MgCl_2_) and calcium chloride (CaCl_2_) can be used as substitutes for NaCl to strengthen hydrophobic interactions, weaken hydrogen and electrostatic interactions, and improve the gel strength of myofibrillar proteins. However, reducing sodium salt decreases the ionic strength of muscle, which lowers the solubility of myofibrillar proteins and leads to a decline in the water-holding capacity and gel-forming ability of meat products [20]. To address these challenges, studies have shown that basic amino acids such as arginine (Arg) and L-lysine (Lys) can alter the structure of myosin, enhance protein solubility, and mitigate the negative effects of metal substitute salts on meat products [21]. Additionally, polyhydroxy alcohol lowers the final salt content of meat products by reducing water activity and slowing salt diffusion, while preserving edible quality and inhibiting microbial growth [22].

Marinated beef is a common cooking ingredient in China. Aiming to reduce sodium content while maintaining product quality, our research developed a compound low-sodium salt substitute (CLSAS) [23] containing K lactate, MgCl_2_, Ca ascorbate, lysine, and sorbitol. Static marinating is a widely adopted method, but it has limitations in efficiency and uniformity, particularly when applied in large-scale or continuous production [24]. These shortcomings highlight the need for alternative techniques to optimize the marination process. Vacuum tumbling [25,26] is a widely adopted method in the meat industry. By combining a vacuum environment with mechanical action, this technique enhances the penetration of the marinade into muscle tissue and promotes effective interactions between the marinade and proteins.

Therefore, this study proposes utilizing vacuum tumbling technology in combination with CLSAS to produce marinated beef. The effects on the meat’s physicochemical properties, water distribution, and microstructural characteristics were evaluated. This study aims to provide a reference for the development of low-sodium meat products by combining CLSAS with vacuum tumbling technology in order to promote its successful application in industrial production.

## 2. Materials and Methods

### 2.1. Marinated Beef Preparation

Chilled beef knuckle, harvested within one day postmortem, was purchased from a local supermarket, packaged in an ice box, and transported to the laboratory within one hour. NaCl, K-Lactate, MgCl2, Ca-Ascorbate, Lys, and sorbitol were all food graded (Shanghai Jinjing Chemical-engineering Co., Ltd., Shanghai, China). All chemicals were purchased from Solarbio Corporation (Beijing, China).

Three independent batches of beef were prepared, each batch containing three beef knuckles. The muscle was trimmed of fascia and visible fat, sliced (35 mm × 25 mm × 8 mm, length × width × height) along the muscle fiber direction with a slicing machine, vacuum packed, frozen at −18 °C until use, and then divided into approximately 120 slices from the three knuckles. After being thawed at 4 °C overnight, these slices were randomly divided into three equal groups equally and immersed in the marinade solution, as shown in Table 1. F and L were statically marinated for 4 h at 4 °C, with the meat slices turned every hour to ensure even marination. VT-L was marinated using a vacuum tumbler (GR-30, Zhucheng Hengshun Machinery Co. Ltd., Zhucheng, China) for 3 h (tumbled for 30 min, intermittent for 10 min) at 4 °C, 0.06 MPa, 10 r/min.

### 2.2. Product Yield and Cooking Loss

The product yield (PY) in this study was determined by measuring the weight differences of the beef before and after the marinating process. The initial weight of beef before marinating (*w*_1_) and the final weight of marinated beef after marinating (*w*_2_) were recorded in grams (g). Prior to measurement, excess surface liquid was removed by lightly draining the marinated beef. The product yield was calculated using the following formula, as described below:

PY (%) = *w*_2_/*w*_1_ × 100% (1)

The cooking loss (CL) of the marinated beef in this study was determined using the following method [27]. Ten pieces of beef were randomly selected from each group, weighed (*w*_2_), and put separately into cooking bags. Thermocouples were placed in the center of each sample to monitor the internal temperature. The samples were heated in a water bath (78 °C) and the whole bag was taken out when the core temperature of the meat reached 72 °C. After cooling to room temperature (25 °C) by flushing the bag with tap water, the meat was taken out and its surface moisture was wiped off with absorbent paper. Then, the cooked marinated beef was reweighed (*w*_3_), and the cooking loss was expressed as a percentage of the meat’s weight before and after cooling. The calculation for determining the cooking loss was as follows:

CL (%) = (*w*_2_ − *w*_3_)/*w*_2_ × 100% (2)

### 2.3. Texture Profile Analysis and Shear Force

A texture analyzer (TMS-PILOT, Food Technology Co., Sterling, VA, USA) was employed for texture profile analysis (TPA) and shear force determination. After cooking, each marinated beef sample was precisely cut into pieces (10 mm × 10 mm × 8 mm). These pieces were then positioned beneath a 35 mm diameter cylindrical probe at a height of 15 mm and compressed twice to 50% of their original height. Force-by-time deformation curves were plotted at a crosshead speed of 50 mm/min and with a trigger force of 0.75 N. Data regarding hardness (N), springiness (mm), cohesiveness, and chewiness (mj) values were collected and subsequently analyzed. Ten samples were analyzed for each group.

To assess the shear force, the cooked beef was prepared as per the instructions described by Biffin et al. [28] and chopped into uniform pieces of 25 mm × 10 mm × 8 mm. A texture analyzer with a probe (HDP/BS) was used to detect the shear force at the same position in each sample. The probe was lowered to a depth of 25 mm, and the sample was sheared laterally at a constant speed of 50 mm/min with a trigger force of 0.75 N. The maximum peak force recorded during the test was reported as the shear force value. Ten samples were analyzed for each group.

### 2.4. Color

The color of the marinated beef was analyzed utilizing an ADCI series automatic colorimeter (Beijing Chentec Instrument Technology Co. Ltd., Beijing, China) with D65 standard illuminant, 10° standard observer, and a 15 mm aperture. The average value of three separate color measurements was recorded, and the outcomes were quantified based on lightness (L*), redness (a*), and yellowness (b*). Ten samples were analyzed for each group.

### 2.5. Water Activity and Water Content

The water activity (*Aw*) of the samples were measured according to the method by Yang [23], using a water activity meter (Changer Tek Industrial (Shanghai) Co., Ltd., Shanghai, China). The water content (WC) was determined using a moisture analyzer (MA 37, Sartorius, Germany). Briefly, a 2 g sample was cut off from the center of the meat slice and then heated up to 105 °C until constant weight was reached. WC was calculated from the difference in the weight before (*w*_4_) and after heating (*w*_5_):

WC (%) = (1 − *w_5_*/*w_4_*) × 100% (3)

### 2.6. Low Field Nuclear Magnetic Resonance (LF-NMR) Analysis and Magnetic Relaxation Image (MRI) Measurements

An NMR analyzer (Meso MR20-0660H-I, Niumag Electric, Shanghai, China) was used to analyze the changes in moisture status of the marinated beef. Approximately 4 g of sample was placed in a 6.0-diameter tube and inserted into the NMR probe. The Carr–Purcell–Meiboom–Gill (CPMG) method was employed to measure the transverse relaxation time. The pulse parameters were set as follows: main frequency = 21.00 MHz, migration frequency = 200 kHz, pulse width at 90° (P1) = 13.00 μs, pulse width at 180° (P2) = 26.48 μs. The relaxation measurements were performed at the optimal operating temperature of 32 °C.

The marinated beef samples were also scanned using an NMR analyzer with a spectrometer frequency of 21.05 MHz and an operating temperature of 32 °C. The proton density images were acquired using an SE imaging sequence with a repetition time (TR) of 300 ms and an echo time (TE) of 15.0 ms. The samples were divided into five layers for analysis, with a slice thickness of 5.0 mm and a slice gap of 1.11 mm. All images were acquired at a position of half the height of the meat samples. The distribution of proton density in the images was obtained using the embedded image processing software.

### 2.7. Fourier Transform Infrared (FT-IR) Spectroscopy

Changes in functional groups and protein secondary structure in the samples were analyzed via FT-IR. Prior to the analysis, the beef samples were subjected to lyophilization for 40 h in a freeze dryer (FDU-1200, STIK, Shanghai, China) and then stored in a desiccator until further use. Pellets were prepared by blending the dried samples with KBr (1:100, *w*/*w*). Then, the infrared spectrum was measured with an FT-IR spectrometer (VERTEX 70v, Bruker, Berlin, Germany) in the range of 4000 cm^−1^ to 400 cm^−1^, with a resolution of 4 cm^−1^, 16 scanning times.

### 2.8. Histological Analysis

Samples were subjected to treatment in a vessel with transverse fiber directions. Tissues obtained from the surface of the treated sample underwent fixation for 48 h in a solution of 10% phosphate-buffered formaldehyde (pH 6.9–7.1). Tissue blocks measuring 10 mm × 10 mm × 5 mm were excised from the fixed meat samples and were then dehydrated overnight using an automatic tissue processor (Donatello Series 2, Diapath S.p.A., Martinengo, Italy). The dehydrated tissue blocks were subsequently embedded in paraffin wax and cut into 4 μm thick sections using a rotary microtome (Microsystems RM2016, Leica, Wetzlar, Germany). The sections were dried overnight at 60 °C and stained with hematoxylin and eosin. The stained sections were analyzed using an optical microscope (DMi1 biomicroscope, Leica, Wetzlar, Germany), and images were captured using a digital video camera (Imaging micropublisher 3.3 RTV, Surrey, Canada). Image J software (v1.8) was employed to measure the diameter, area, and density of muscle fibers in each microscope image.

### 2.9. Statistical Analysis

Three separate batches of marinated beef were prepared, with each batch prepared using a total of three treatments and replicated under corresponding conditions. Measurements were taken at least three times for each sample. Data were evaluated using a general linear model (GLM), with marinating method (F, L, and VT-L) considered a fixed effect and the replicate as a random effect. All data were expressed as the means ± standard error. The differences between means of values among the three different treatments were assessed by one-way analysis of variance (ANOVA) using the SPSS Statistics package (v20.0, SPSS Inc., Chicago, IL, USA). Significant differences (*p* < 0.05) between means were identified using Duncan’s multiple range test.

## 3. Results and Discussion

### 3.1. Product Yield and Cooking Loss

**Figure 1:**
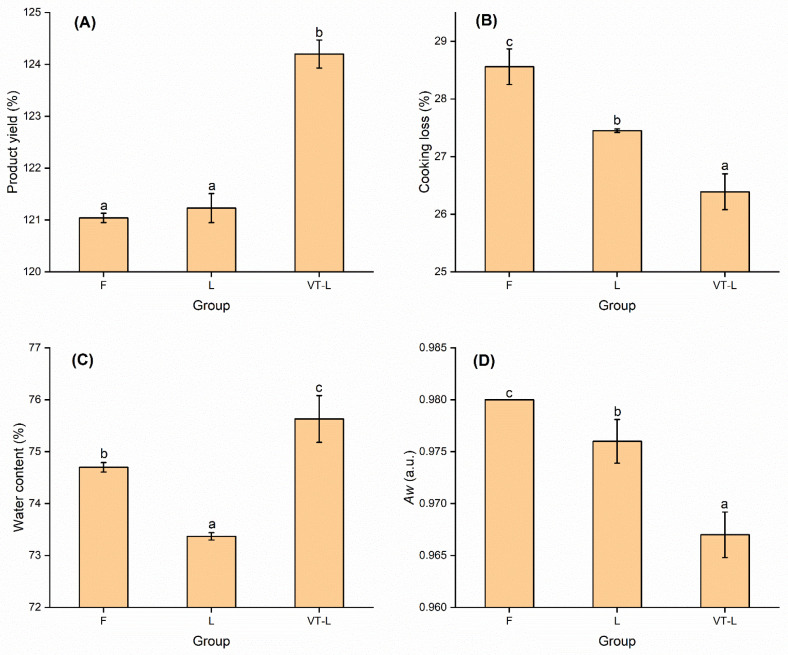
Product yield (**A**), cooking loss (**B**), water content (**C**), and *Aw* (**D**) of marinated beef slices after full-sodium static pickling (F), compound alternative salts static pickling (L), and compound alternative salts vacuum tumbling (VT-L). a–c: Mean ± standard error in the same column with different superscripts means significantly different at α = 0.05.

PY is a crucial parameter in the meat industry, directly impacting economic benefits. The data presented in Figure 1A demonstrate that VT-L had a significant effect on enhancing product yield (*p* < 0.05). Compared with static marination, the increase in PY observed in VT-L can be attributed to the vacuum effect, which facilitated rapid penetration by the marinade solution. U-Chupaj et al. [29] found that vacuum-tumbling marination could enhance chicken breast PY. This, in turn, led to a more uniform absorption of the solution and minimized water loss [30]. Additionally, the continuous tumbling of the beef in the barrel via wrestling and massaging actions resulted in loosening of the muscle tissue, dispersion of the myofibrils, softening of the connective tissue, and increased contact between proteins and the marinade solution [31,32].

Water-holding capacity refers to a protein’s ability to retain water under various conditions and is closely linked to the behavior of meat during cooking, with cooking loss serving as an indicator of water-holding capacity [33]. Salt has been shown to aid in the extraction of myofibrillar protein and enhance water-holding capacity. However, reducing sodium levels can negatively impact the muscle’s ability to retain water [34]. Bower et al. [35] demonstrated that the water-holding capacity of roast beef decreased as the concentration of NaCl reduced, resulting in lower product yield and higher cooking losses. In our experiment, the product yield of groups F and L under static pickling conditions was similar. However, the CL of group L was lower than that of group F, suggesting that the CLSAS formulation used in the low-sodium salt-marinated beef helped retain the marinade and reduce cooking losses (Figure 1B). This improvement may be attributed to the CLSAS formulation containing MgCl_2_. The presence of divalent Mg^2+^ increased the solubility of myosin, allowing salt-soluble myogenic fibrin to bind more free water during thermal gel formation, so that cooking losses were reduced [36]. Furthermore, the addition of Lys to the marinated beef raised the pH value and caused the pH of salt-soluble protein in the muscle tissue to deviate from its original isoelectric point [37,38]. This increased intramolecular electrostatic repulsion and enhanced the interaction between proteins and water, ultimately improving the water-holding capacity of the muscle [39]. VT-L exhibited the lowest cooking losses, which was likely to have been due not only to the effect of MgCl_2_ and Lys but also the vacuum environment and mechanical beating during the tumbling process, which enhanced the accumulation of salt-soluble proteins on the meat surface, thereby improving its water-holding capacity and reducing outward diffusion [40].

Overall, the vacuum-tumbling treatment and the CLSAS formulation significantly improved the water-holding capacity of the myogenic fibers and increased the product yield of meat products.

### 3.2. Water Content and Water Activity

VT-L led to a significant increase (*p* < 0.05) in WC in the marinated beef (Figure 1C). N’Gatta [41] observed higher WC in tumbled beef compared with un-tumbled. These results indicated that the application of vacuum tumbling treatment that involved the squeezing and pulling of the beef facilitated the ingress of moisture into the meat product. Moreover, the WC in L was lower than that in F, possibly due to the presence of Ca^2+^ and Mg^2+^ reducing the repulsive forces between proteins. This reduction in repulsive forces limits the adsorption of water molecules onto the surface of myofilaments [42].

*Aw* exhibited significant differences (*p* < 0.05) among the three groups, with VT-L < L < F (Figure 1D). This observation suggests that the CLSAS formulation and vacuum-tumbling treatment can effectively decrease the *Aw* of meat products. Moreover, the inclusion of sorbitol in the marinade has been found to be particularly effective in reducing *Aw*, probably due to its capacity to bind water molecules [43]. This binding action restricts the mobility of water within the marinated beef. Consequently, vacuum tumbling not only enhances the binding of water to myogenic fibers but also improves the water-binding capacity of meat products.

### 3.3. Color

**Figure 2:**
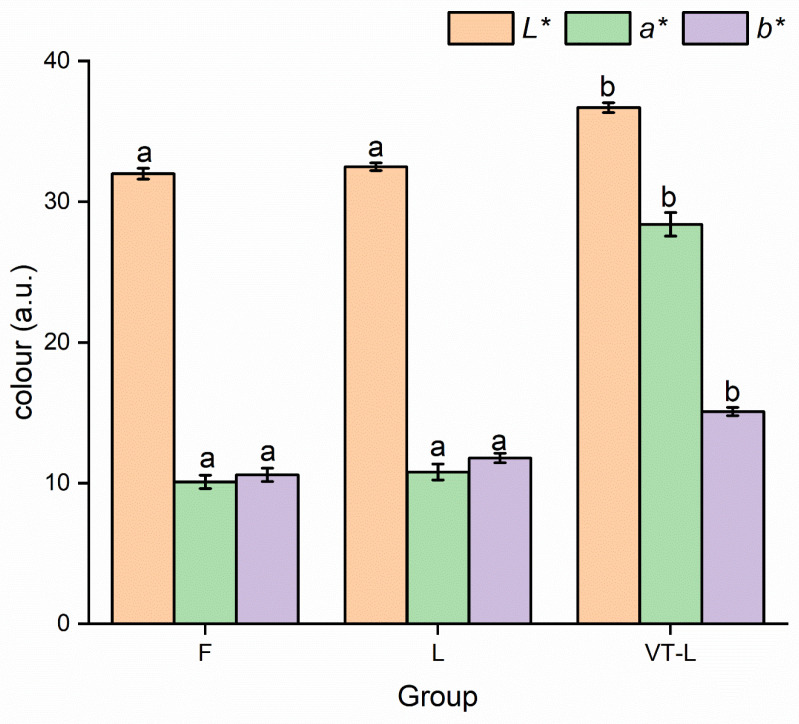
Color of marinated beef slices after full-sodium static pickling (F), compound alternative salts static pickling (L), and compound alternative salts vacuum tumbling (VT-L). a–b: Mean ± standard error in the same column with different superscripts means significantly different at α = 0.05 level.

Color is an essential visual attribute of marinated beef, directly impacting consumer acceptance [44]. Figure 2 revealed that there were no statistically significant discrepancies in color variation among the samples within groups F and L. This observation suggested that the implementation of the CLSAS did not exert adverse effects on the coloration of the marinated beef. However, the vacuum-tumbling treatment significantly enhanced the color of the meat. The values of L*, a*, and b* in VT-L were significantly higher than those in both F and L (*p* < 0.05). The bright color of the meat may have been due to its higher moisture content causing surface light reflection and thus, a higher L* value [45]. Uyarcan et al. [46] also found similar results in marinated beef loin steaks, where the high L* values of the samples were potentially attributable to the denaturation of myoglobin and the displacement or release of heme groups in the meat.

The a* value in the VT-L group showed a significant difference compared with the other two groups (*p* < 0.05). The color of marinated beef became dark salmon (higher a*) after the vacuum-tumbling treatment. This may be attributable to the transformation of oxymyoglobin (bright red) or metmyoglobin (light brown) to myoglobin (purplish red) in a low-oxygen environment under the influence of vacuum [47].

### 3.4. Texture Profile Analysis and Shear Force

Table 2 shows that the CLSAS and vacuum-tumbling treatment primarily affected the hardness and chewiness of the marinated beef. Significantly lower values for these two attributes were observed in L and VT-L compared with F (*p* < 0.05), while other textural profiles like cohesiveness and springiness showed no significant differences (*p* > 0.05). Samples marinated in high-salt solutions undergo important protein denaturation, resulting in a harder texture [48]. Li et al. [37] found that the replacement of NaCl had no negative impact on the textural profile of beef, and the current study had a similar result. Moreover, the disruption of cell membranes throughout the muscle during the tumbling process [49] led to a reduction in the thickness of both outer and inner muscle membranes, subsequently resulting in decreased hardness of the meat [50].

Samples in the VT-L group exhibited the lowest shear force value in this study (Table 2). Vacuum tumbling has been demonstrated to enhance the tenderness of marinated beef, as it promotes the degradation or damage of proteins under mechanical force [51]. Additionally, the decrease in shear force could be associated with chemical changes induced by the absorption of marinade components into the loose myogenic fiber structure. Research has shown that the content and concentration of organic acid-based or salt-based marinade liquids significantly impact the recovery of muscle tenderness during processing [52]. The shear force value in group L was significantly lower than that in group F (*p* < 0.05), which is likely to have been due to the activity of calpain, which was activated by the addition of Ca ascorbate [53]. This enzyme acceleration promotes the degradation of troponin-T, thereby improving tenderness [54].

In addition, sorbitol can act as a moisturizing agent and improve water-retention capacity and reduce shear values at higher concentrations [55]. Consequently, both vacuum tumbling and the low-sodium alternative salts compound exerted an increased effect on the marinated beef’s tenderness through both physical and chemical pathways, thereby improving its textural characteristics.

### 3.5. LF-NMR and MRI

**Figure 3:**
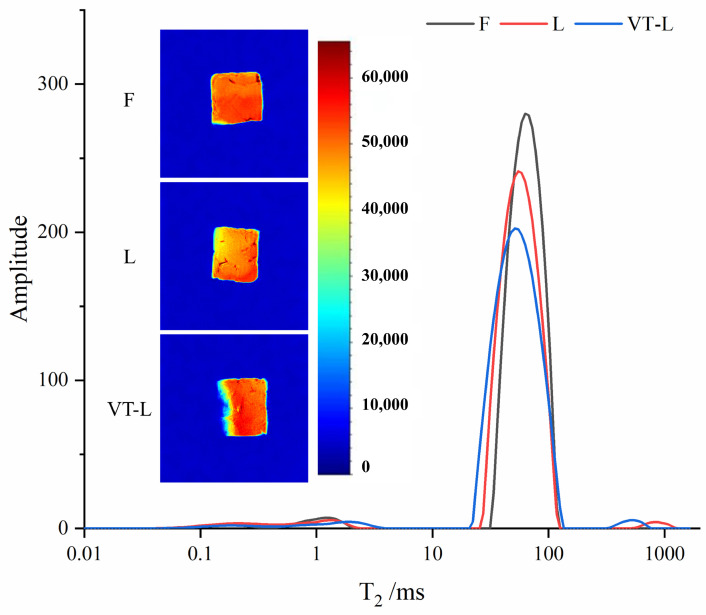
T_2_ relaxation spectrum and magnetic resonance imaging (MRI) of marinated beef after full-sodium static pickling (F), compound alternative salts static pickling (L), and compound alternative salts vacuum tumbling (VT-L).

The LF-NMR T_2_ relaxation curves obtained from the marinated beef samples displayed a distribution characterized by multiple-exponential decay. Group F exhibited three distinct populations, whereas group L and group VT-L displayed four, primarily influenced by the water state (Figure 3). These were identified as T_20_ (0.1–1 ms), T_21_ (1–10 ms), T_22_ (30–100 ms), and T_23_ (>100 ms) [25,50].

Generally, lower T_2_ indicates a tighter bond between the marinated beef and the water molecules in this state [56,57]. Table 3 reveals that there was no significant difference among the three groups for T_20_ and T_21_ (*p* > 0.05), indicating the stability of bound water in these samples. There was also no significant difference between L and VT-L (*p* > 0.05), but the T_22_ of VT-L was lower than that of L. It has been established that the tumbling action can enhance the water content of meat products by altering water distribution. Vacuum tumbling also modifies the spatial structure of myofibrillar proteins, potentially exposing polar hydrophilic groups within the proteins and ultimately aiding water retention [58]. Meanwhile, the T_22_ in L was slightly lower than that in F, possibly due to the presence of divalent cations (such as Ca^2+^ and Mg^2+^) in the low-sodium alternative salt formulation, affecting myofibrillar protein cross-linking and reducing drip loss as a result [59]. The presence of a T_23_ peak was observed only in group L and group VT-L, with a significantly lower value in the latter group (*p* < 0.05). This may be attributed to the reduced NaCl content, which enhanced water mobility in these two groups. A decrease in free water (T_23_) is linked to an increase in muscle water-retention capacity [60]. Vacuum-tumbling treatment improved muscle binding to water, as evidenced by the movement of T_23_ in the VT-L group towards a lower T_2_ value compared with group L. The relaxation properties of T_22_ and T_23_ are both associated with myogenic fiber swelling and are considered indicators of the geometric shape of myogenic fibers [61]. The reduction in T_22_ and T_23_ after tumbling treatment can be attributed to decreased water mobility or increased interaction between water and the myofibrillar protein structure, indicating the expansion of the marinated beef due to the vacuum-tumbling treatment. In conclusion, the combination of a low-sodium alternative salt and vacuum tumbling effectively enhanced the water-holding capacity of marinated beef.

The distribution of H protons inside the material under examination was visualized using MRI, where the intensity of darker red indicated a higher density of H protons and darker blue represented a lower density of H protons [62]. As depicted in Figure 3, VT-L exhibited a more pronounced and homogeneous distribution of redness in the central region compared with those in F and L, suggesting a higher content of H protons, indicating a higher moisture content resulting from vacuum tumbling. This finding is consistent with the T_2_ value and *Aw* results. However, the left half-edge section of the beef in group VT-L displayed a lighter color, which may be attributed to excessive disruption at the muscle tissue edge due to the pulling and kneading during vacuum tumbling, resulting in partial loss of moisture.

### 3.6. FT-IR

**Figure 4:**
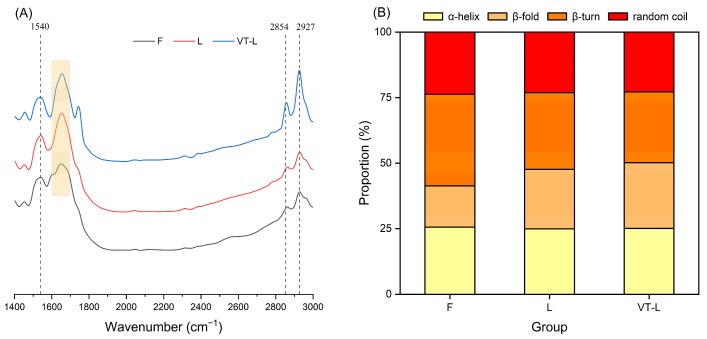
Infrared spectrum at 1400–3000 cm^−1^ (**A**) and protein secondary structure (**B**) of marinated beef after full-sodium static pickling (F), compound alternative salts static pickling (L) and compound alternative salts vacuum tumbling (VT-L).

Figure 4A presented the infrared spectrum of marinated beef. The amide II absorption band appeared at 1540 cm^−^^1^, while the symmetric and asymmetric stretching vibrations of methylene (CH_2_) and methyl (CH_3_) groups were observed at 2854 cm^−^^1^ and 2927 cm^−^^1^, respectively [63,64]. As shown in Table 4, the absorbance of VT-L at 1540 cm^−^^1^ was significantly higher than that of F and L, probably because the vacuum tumbling enhanced protein solubility and the unfolding of aggregated protein structures, consistent with the findings by Zhu et al. [58]. In the 2854 cm^−^^1^ and 2927 cm^−^^1^ bands, the absorbance of VT-L and L was significantly lower than that of F, suggesting increased conformational order and flexibility of protein subunits in these groups [65]. This indicates that both CLSAS and vacuum tumbling can alter protein conformation and influence the degree of protein folding.

The amide I band (1600–1700 cm^−^^1^) reflects C=O stretching in the peptide backbone and is commonly used to analyze protein secondary structure [66]. Curve fitting was used to determine the sub-peak areas of the amide I band (Figure 4B). Protein integrity is indicated by the α-helix and β-sheet, while the random coil and β-turn suggest structural randomness. A significant decreasing trend in the relative content of β-turn from group F and group L to group VT-L was observed, while the relative content of β-fold showed a significant increasing trend (*p* < 0.05). This indicates that the CLSAS and vacuum-tumbling treatment promoted the β-turn to β-fold transformation in the protein secondary structure, resulting in a relatively more ordered network structure and improved structural stability of the protein [67].

### 3.7. Histological Analysis

**Figure 5:**
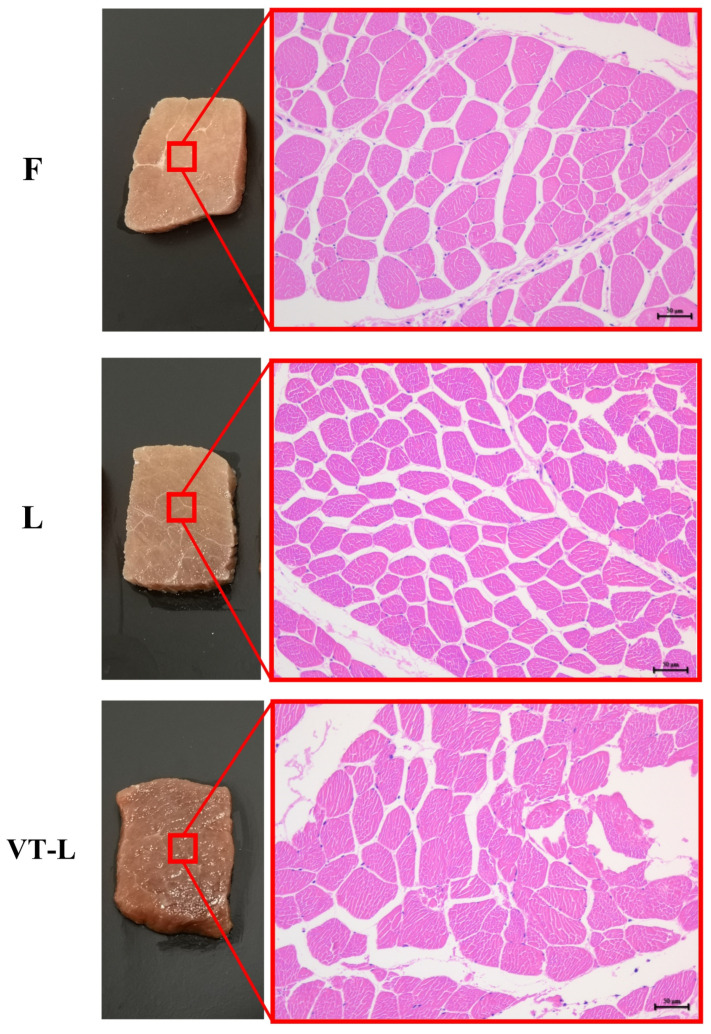
Optical photographs (left) and partial microscope imaging (right, ×200) of the marinated beef samples after full-sodium static pickling (F), compound alternative salts static pickling (L), and compound alternative salts vacuum tumbling (VT-L).

Microstructural changes in the muscle tissue of meat products provide insights into their texture and water-holding capacity. Figure 5 shows the appearance of treated samples from three distinct groups, observed under an optical microscope at 200 magnification. In comparison to group F, group L, which underwent pickling with CLSAS, exhibited clear differentiation between myogenic fibers and sarcolemma. On the other hand, in the VT-L group characterized by the vacuum-tumbling treatment, the myogenic fibers appeared swollen, deformed, and compressed against one another. Additionally, the boundary between the myogenic fibers and sarcolemma became indistinguishable, and individual myogenic fibers exhibited signs of dissolution and breakage, consistent with findings in prior studies [68].

The tissue characteristics of myofibrils are presented in Table 5. Group F exhibited the highest area of myofibrils, attributed to the elevated Na^+^ concentration in this group that enhanced water absorption by myofibrillar proteins. As a result, there was an increase in both cross-sectional area and myofibril diameter [69]. On the other hand, group L had a lower area of myofibrils, probably due to its lower sodium content and less advanced state of myofibril degradation. However, it displayed the highest density of muscle fiber. A study conducted by Fahey et al. [70] on lamb muscle discovered a significant positive correlation between muscle fiber density and meat quality. This factor may explain why L had lower hardness than F. Furthermore, the vacuum-tumbling treatment improved the degradation of myofibrils and reduced the extracellular space. Consequently, the sample from the VT-L group exhibited a significant increase in myofibril area and a slight increase in myofibril diameter compared with L.

Soji [71] investigated meat’s tenderness by analyzing its muscle nanostructures and discovered that tenderness had an inverse correlation with muscle fiber diameter. Consequently, this may also be one of the reasons for the enhanced tenderness observed in marinated beef from group L and group VT-L in comparison to that from group F. The tumbling process was shown to have the ability to disrupt both the superficial and underlying cell membranes of muscles, leading to increased tenderness [40]. Figure 5 illustrates that the boundary between individual muscle fibers and the sarcolemma in meat from group VT-L was relatively fuzzy. Furthermore, the sarcolemma was severely damaged, indicating that the altered tenderness of the marinated beef in group VT-L was attributable to damage to the muscle fiber and connective tissue caused by the vacuum-tumbling treatment.

## 4. Conclusions

The use of CLSAS can maintain the product yield and color of marinated beef comparable to 4% NaCl, while reducing cooking loss, hardness, and shear force. When combined with vacuum tumbling, CLSAS further improved product yield, texture, and color, while enhancing moisture content and reducing cooking loss, *Aw*, and shear force. CLSAS in combination with vacuum tumbling reduced the relaxation time of immobilized water, enhanced the water-holding capacity of myogenic fibers, and increased protein structural order, leading to changes in microstructure.

These improvements in tenderness, water distribution, and overall product quality highlight the potential of CLSAS combined with vacuum tumbling for practical application in low-sodium meat production, providing a viable path for eventual industrial-scale use.

## Figures and Tables

**Table 1:** Marinade solution for the three tested groups. The percentage refers to the ratio of the ingredient to the curing liquor, and “-” means not added.

Group	Solid–Liquid Ratio	Marinade Ingredients/%					
		NaCl	K-Lactate	MgCl_2_	Ca-Ascorbate	Lys	Sorbitol
F	1:2	4	-	-	-	-	-
L	1:2	2.4	0.8	0.4	0.4	0.2	4
VT-L	10:3	2.4	0.8	0.4	0.4	0.2	4

**Table 2:** Textural profile and shear force of marinated beef slices after full-sodium static pickling (F), compound alternative salts static pickling (L), and compound alternative salts vacuum tumbling (VT-L).

Groups	Hardness/N	Cohesiveness	Springiness/mm	Chewiness/mj	Shear Force/N
F	30.2 ± 0.04 ^c^	0.5 ± 0.00 ^a^	1.7± 0.03 ^a^	24.0± 0.36 ^c^	47.6 ± 0.46 ^a^
L	21.3 ± 0.72 ^b^	0.5 ± 0.02 ^a^	1.9 ± 0.09 ^a^	18.4 ± 0.55 ^b^	45.1 ± 0.46 ^a^
VT-L	17.9 ± 0.27 ^a^	0.4 ± 0.01 ^a^	1.8 ± 0.10 ^a^	14.3 ± 0.97 ^a^	39.7 ± 0.19 ^b^

a–c Mean ± standard error in the same column with different superscripts means significantly different at α = 0.05.

**Table 3:** Transverse relaxation time (T_2_) values of marinated beef after full-sodium static pickling (F), compound alternative salts static pickling (L), and compound alternative salts vacuum tumbling (VT-L).

Groups	T_2_/ms			
	T_20_	T_21_	T_22_	T_23_
F	0.68 ± 0.12 ^a^	1.17 ± 0.12 ^a^	58.87 ± 4.08 ^b^	-
L	0.19 ± 0.02 ^a^	1.34 ± 0.05 ^a^	54.79 ± 0.00 ^ab^	821.43 ± 0.00 ^b^
VT-L	0.43 ± 0.18 ^a^	1.73 ± 0.30 ^a^	51.11 ± 0.00 ^a^	554.57 ± 12.98 ^a^

a, b: Means ± standard error in the same column with different superscripts indicate significantly different at α = 0.05; “-” denotes undetectable.

**Table 4:** Absorbance in selected regions of the FT-IR spectrum of marinated beef after full-sodium static pickling (F), compound alternative salts static pickling (L), and compound alternative salts vacuum tumbling (VT-L).

Groups	Absorbance/a.u.		
	1540 cm^−1^	2854 cm^−1^	2927 cm^−1^
F	0.687 ± 0.004 ^b^	0.807 ±0.004 ^b^	0.749 ± 0.004 ^b^
L	0.663 ± 0.009 ^a^	0.789 ± 0.005 ^b^	0.722 ± 0.008 ^b^
VT-L	0.717 ± 0.005 ^c^	0.748 ± 0.007 ^a^	0.634 ± 0.006 ^a^

a–c: Means ± standard error in the same column with different superscripts are significantly different at α = 0.05 level.

**Table 5:** Histological characteristics of the marinated beef samples after full-sodium static pickling (F), compound alternative salts static pickling (L), and compound alternative salts vacuum tumbling (VT-L).

Groups	Muscle Fiber Area/μm^2^	Muscle Fiber Diameter/μm	Muscle Fiber Density/μm
F	3695.94 ± 44.73 ^a^	7.98 ± 0.07 ^b^	110.00 ± 3.89 ^b^
L	2351.85 ± 33.58 ^b^	6.83 ± 0.08 ^a^	153.75 ± 2.10 ^c^
VT-L	3282.96 ± 63.08 ^a^	7.15 ± 0.15 ^a^	80.00 ± 1.15 ^a^

a–c: Means ± standard error in the same column with different superscripts are significantly different at α = 0.05.

## Data Availability

The original contributions presented in the study are included in the article. Further inquiries can be directed to the corresponding author.
